# Cytological diagnosis of patients with embryonal rhabdomyosarcoma of the cervix: case report and literature review

**DOI:** 10.1186/s13000-024-01497-y

**Published:** 2024-06-03

**Authors:** Xiaoxia Wei, Lei Li

**Affiliations:** 1 Department of Pathology, West China Second University Hospital, Sichuan University, Chengdu, China; 2 Key Laboratory of Birth Defects and Related Diseases of Women and Children, Sichuan University, Ministry of Education, Chengdu, China

**Keywords:** Uterine cervix, Liquid-based cytology, Cervical embryonal rhabdomyosarcoma

## Abstract

Cervical embryonal rhabdomyosarcoma(ERMS) is a rare malignancy. To date, no cases of ERMS diagnosed by cervical cytology have been reported. In this study, we report a case of cervical ERMS identified by a liquid-based cytology test and cell blocks in a 46-year-old postmenopausal woman. We describe the cytological features of ERMS, with the aim of helping cytopathologists recognize this rare cervical tumor.

## Background

Rhabdomyosarcoma (RMS) is the most common malignant solid tumor in children [1], but RMS of the cervix is a rare disease in adults [2]. RMS can occur in any tissue in the body [3]. RMS occurs most frequently in the genitourinary tract and the head and neck [4]. Embryonal RMS (ERMS) displays the most common histology and can be further classified into the classic subtype, botryoid subtype, and spindle cell subtype [5]. The cytological characteristics of ERMS are not well-defined, and there are very few reports in the literature on the cytology of ERMS. We report the case of a 46-year-old perimenopausal woman diagnosed with ERMS by liquid-based cytology testing, cell block examination and immunohistochemistry. We describe the cytological features of ERMS as well as the cell-block and immunohistochemical findings and demonstrate that a definite diagnosis of ERMS can be made based on cytologic examination alone.

## Case presentation

This 46-year-old female patient presented to a local hospital four months earlier with “intermenstrual bleeding,” and a physical examination revealed a cervical neoplasm. Cervical polypectomy and laser treatment were performed at the local hospital one month later, and the postoperative pathology revealed a polyp (no reports). There were a few recent bloody vaginal secretions, but no other discomfort occurred. The patient reached menarche at age 13. The patient had a total of 9 gestations, including 5 productions and 4 miscarriages.  Gynecological physical examination revealed a 4 × 4 cm cervical neoplasm as well as blood and papilla of the cervical canal. Subsequently, cervical cytology and HPV typing were performed.

**Fig. 1:**
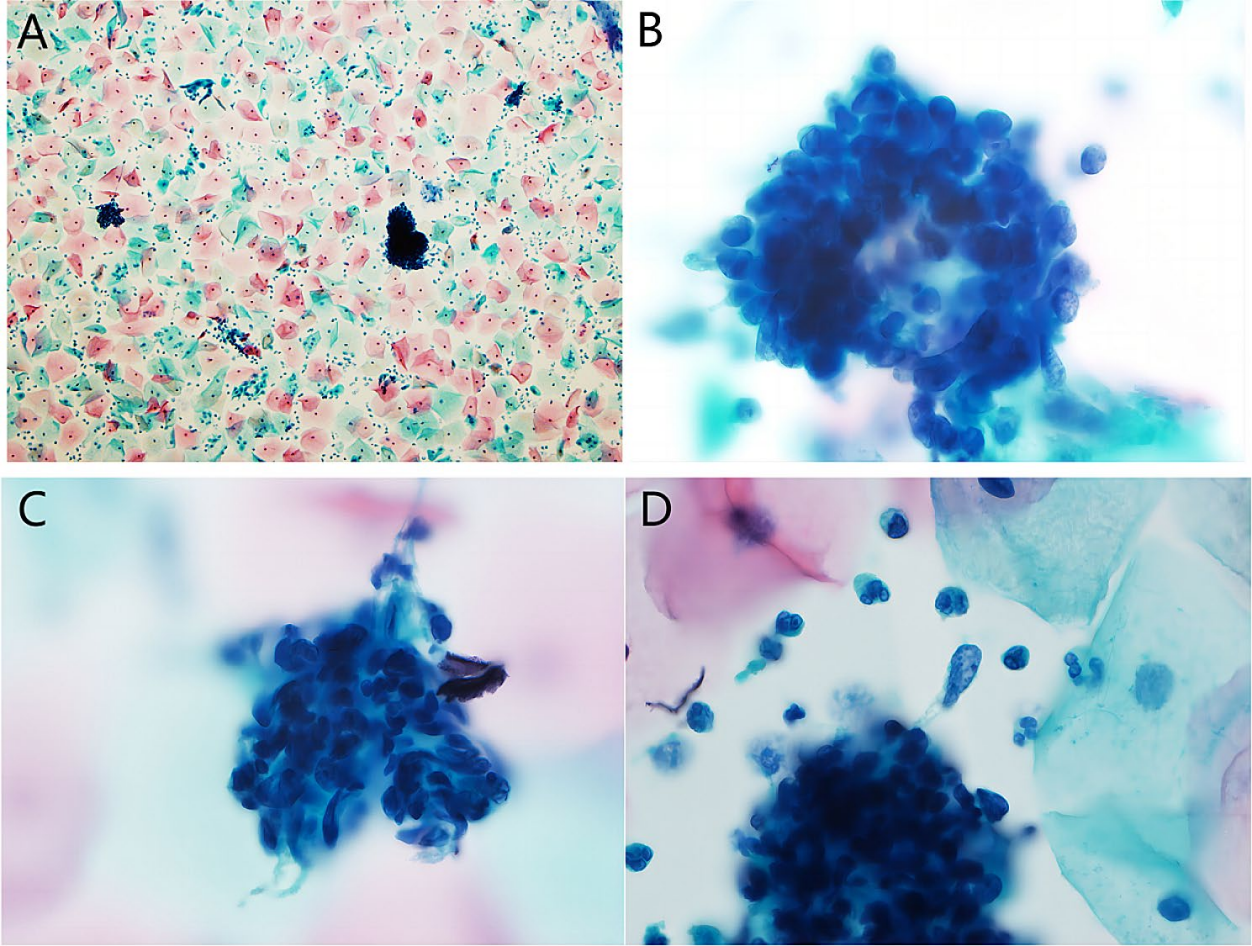
Cervical smear showing small sheets of cells, small clusters of cells, and scattered small blue cells admixed with the normal squamous epithelium under low-power microscopy (**A**, Papanicolaou stain, ×100). Most of the neoplastic cells had round to oval nuclei with high nuclear to cytoplasmic ratio, scant cytoplasm and indistinct cell border. The surface was layered with epithelial cells, and there were dark-stained small blue cells below. (**B**, Papanicolaou stain, ×1000). Most cells were small and blue, with little cytoplasm and irregular nuclei, and individual cells were somewhat tadpole- or ribbon-shaped. The nuclei were round to oval with evenly distributed coarse chromatin and inconspicuous nucleoli, and the cell size was slightly larger than that of normal lymphocytes. (**C&D**, Papanicolaou stain, ×1000)

**Fig. 2:**
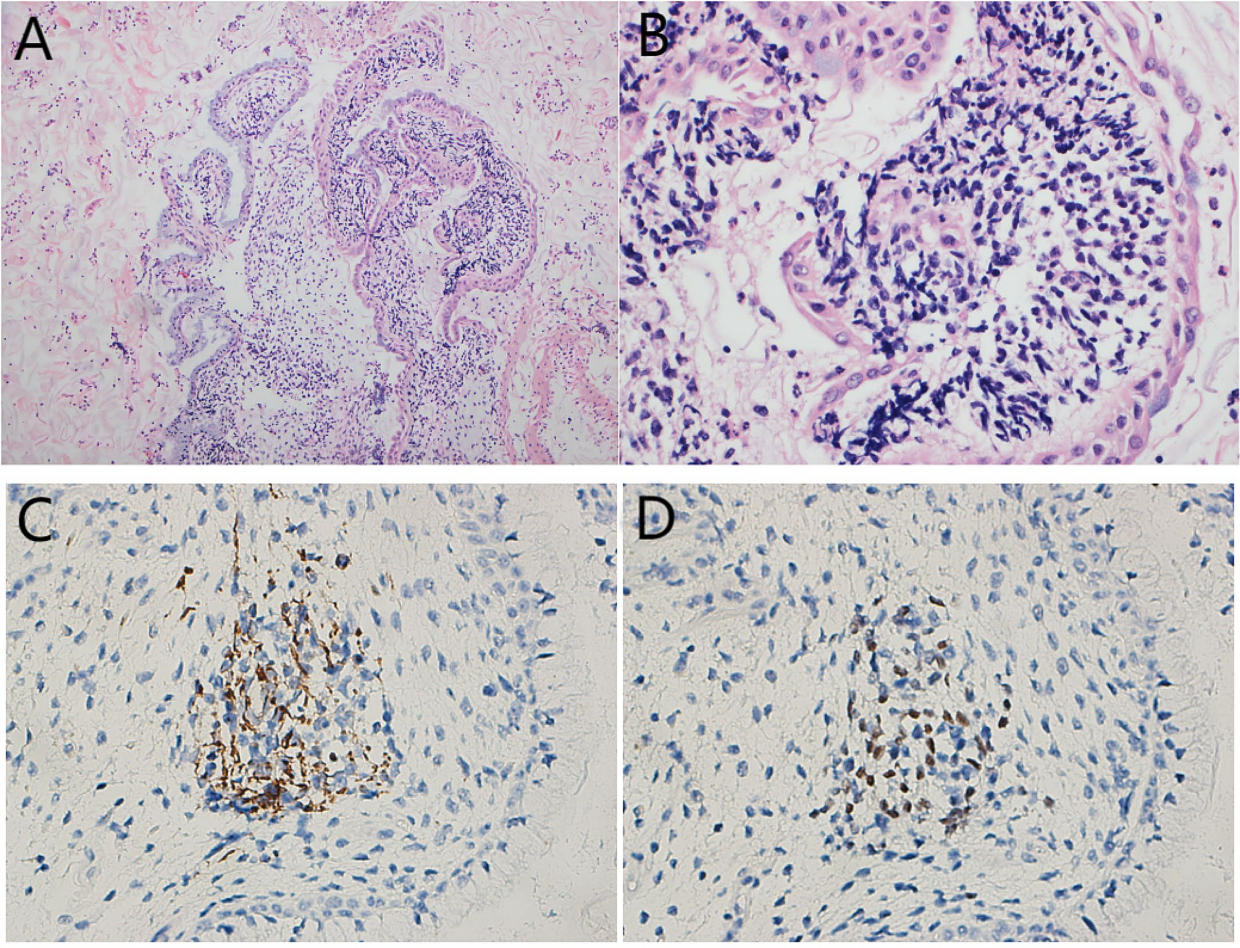
The cell block of the cervical smear showing the examination of polypoid tissue for mucus and inflammatory exudates under low magnification (**A**, H&E, ×100). High magnification revealed that the tissue surface was covered with mucous gland epithelium, and dense small blue cells were distributed band-like under the epithelium. A cambium layer composed of subepithelial condensates of undifferentiated cells was observed. The cells were small and spindle-shaped, and interspersed strap cells were observed (**B**, H&E, ×400). IHC demonstrated that the small blue cells were focally positive for desmin (**C**, ×400**)** and MyoD1 (**D**, ×400)

Then, a cervical smear was performed. Microscopically, we observed some small sheets of cells, small clusters of cells, and scattered small blue cells admixed with the normal squamous epithelium (Fig. 1A). Most of the neoplastic cells had round to oval nuclei with high nuclear to cytoplasmic ratio, scant cytoplasm and indistinct cell border. The surfaces were layered with epithelial cells, and there were dark-stained small blue cells below. Most cells were small and blue, with little cytoplasm and irregular nuclei, and individual cells were somewhat tadpole- or ribbon-shaped. The nuclei were round to oval with evenly distributed coarse chromatin and inconspicuous nucleoli, and the cell size was slightly larger than that of normal lymphocytes (Fig. 1B-D). The initial diagnosis was atypical cells that were considered to constitute a malignant tumor. Then, we made cell blocks from the remaining specimens, and the morphology revealed in the cell blocks was consistent with the cytopathological features of the cervical smear. The cell blocks showed polypoid tissue with mucus and inflammatory exudates under low magnification (Fig. 2A). Examination under high magnification revealed that the tissue surface was covered with mucous gland epithelium, and dense small blue cells were distributed in a band-like manner under the epithelium. A cambium layer was observed, comprising a subepithelial condensation of undifferentiated cells. The cells were small and spindle-shaped, and the strap cells were interspersed (Fig. 2B). On immunohistochemistry (IHC), the small blue cells were focally positive for desmin, MyoD1 (Fig. 2C&D) and myogenin and negative for CK-P, ER and caldesmon. The final diagnosis from cervical cytology was malignant tumor cells predisposed to embryonal rhabdomyosarcoma.

**Fig. 3:**
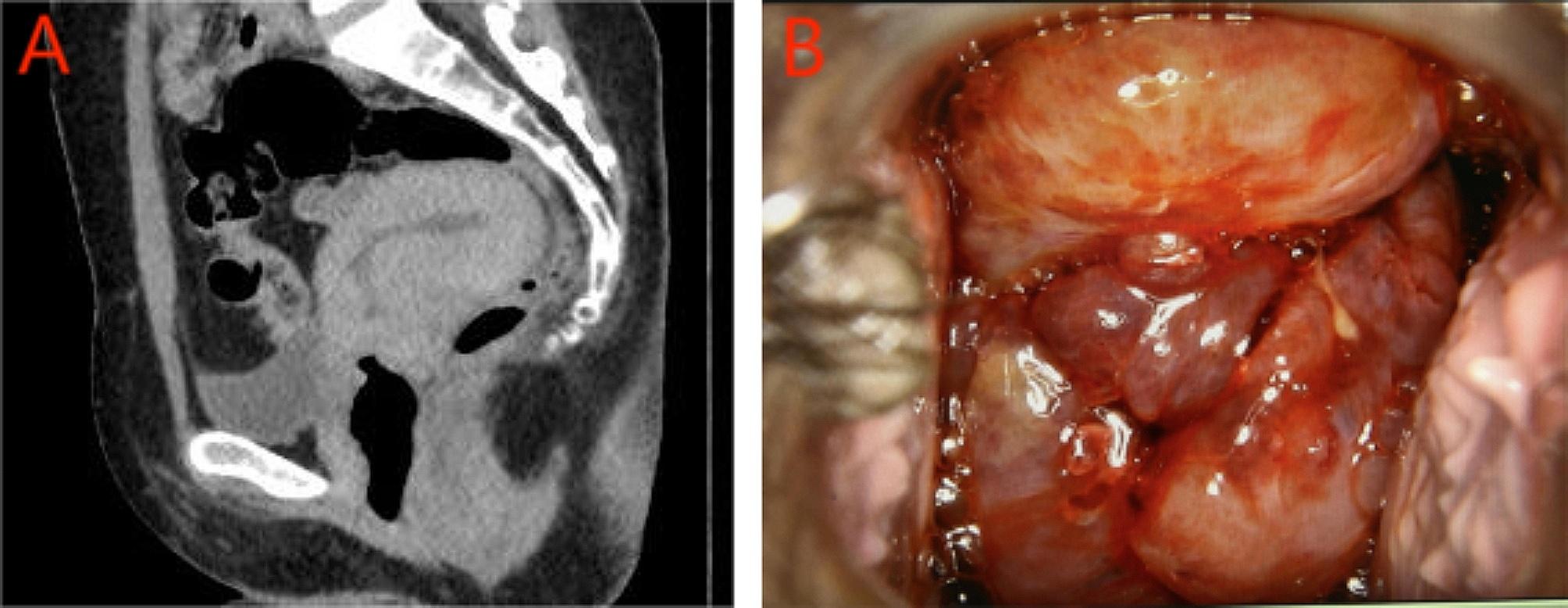
CT revealing an abnormal soft tissue nodule shadow in the cervical canal and an outer cervical opening with a long diameter of approximately 3 cm and uneven enhancement after enhancement (**A**). A cervical lobated neoplasm (4 × 4 cm) was observed via colposcopy. The neoplasm was grape-like in appearance (**B**)

CT revealed an abnormal nodular soft tissue shadow at the cervical canal and outer cervical opening with a long diameter of approximately 3 cm and uneven enhancement (Fig. 3A). A cervical lobated neoplasm measuring 4 × 4 cm in size was observed via colposcopy. The neoplasm had a grape-like appearance (Fig. 3B).

**Fig. 4:**
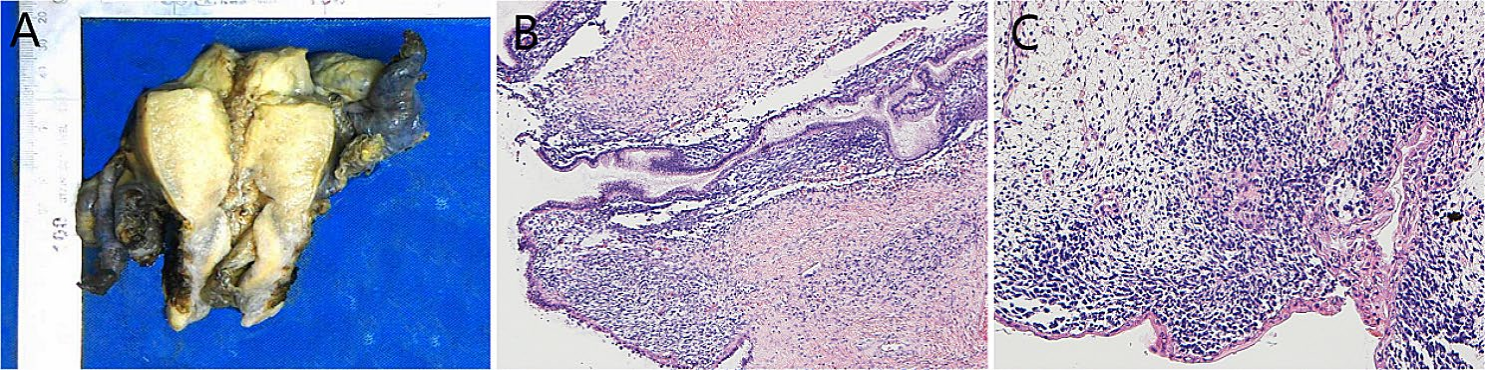
A polyp-like mass measuring 2.7 × 0.6 × 0.5 cm in size observed in the inner mouth of the cervical canal with a wide base (**A**). The tumor was composed of mainly hypocellular areas with rhabdoid changes and myxoid stroma (**B&C**, H&E, ×100&400)

Considering the lack of parametrical and lymph node involvement, a decision was made to proceed with single-port laparoscopic-modified extensive total hysterectomy, bilateral salpingo-oophorectomy, pelvic lymph node dissection and abdominal aortic fat lymph node sampling. A polyp-like mass measuring 2.7 × 0.6 × 0.5 cm in size was observed in the inner mouth of the cervical canal with a wide base. The mass section was grayish-white and soft. The root of the tumor seemed to invade the surrounding tissue (Fig. 4A). The tumor was composed of mainly hypocellular areas with rhabdoid changes and myxoid stroma (Fig. 4B & C). Pathologic examination revealed invasion of the cervical stromal connective tissue. These findings confirmed embryonal rhabdomyosarcoma of the uterine cervix with local invasion into the superficial interstitial layer of the cervical canal. No lymph node invasion was observed. Repeat immunohistochemistry revealed strongly and diffusely positive staining for desmin; focal positive staining for myogenin, MyoD1, myoglobin, CD10 and cyclin D1; and negative staining for CK-P, ER, PR and S-100. The percentage of Ki67-positive cells was approximately 80%. The findings were consistent with a diagnosis of embryonal rhabdomyosarcoma of the cervix. The patient’s disease was classified as FIGO stage IB3 (T1b, N0, M0). Based on the Intergroup Rhabdomyosarcoma Study Group (IRSG) classification, the patient’s case was categorized as IA.

The patient’s postoperative course was unremarkable. The patient subsequently received adjuvant chemotherapy consisting of vincristine, actinomycin-D and cyclophosphamide administered over a 6-month period. Computed tomography (CT) of the abdomen and pelvis after two cycles of chemotherapy did not show any evidence of disease.

## Discussion

Embryonal rhabdomyosarcoma (ERMS) of the cervix is a very rare malignancy, accounting for 0.4-1.0% of all cervical malignancies [4]. The literature reports that up to 90% of cases of ERMS occur in women less than 25 years old, and approximately 60–70% of cases occur in children less than 10 years of age. Occurrence in perimenopausal women is very rare, but the prognosis in this age category is generally poor [6]. Clinical symptoms of ERMS that occur in the female reproductive system often include vaginal bleeding and an exophytic or polypoid mass [5]. The tumor often resembles a grape-like cluster with an average size of 5.75 cm [7]. Among the reported cases, tumors as large as 14 cm have been observed, and the smallest tumor is approximately 1.5 cm in size [7, 8]. The incidence of small round cell tumors such as rhabdomyosarcoma in the cervix is much lower than that of epithelium-derived tumors. In cervical cytology, the morphology overlaps with that of other tumors, so the diagnosis of rhabdomyosarcoma with cervical cytology is very difficult. To date, no cases of cervical ERMS diagnosed by cervical cytology have been reported. Our case was the first reported case involving a diagnosis via liquid-based cytology testing.

The cytopathologic features observed in this patient were as follows: mostly round or oval tumor cells, with high nucleoplasmic ratios, scarce cytoplasm, and indistinct borders. Some of the cells had “naked nuclei” in shape, and some had small clusters or scattered cells that were slightly rich in cytoplasm and had a “drag tail”. The nuclei were round to oval, and a small number of cells were irregular in shape, had deep nuclear staining, were slightly larger than those of normal lymphocytes, had fine chromatin, and had no nucleoli or nuclear divisions. By combining these findings with information from cell block examination and IHC, we finally diagnosed the patient with ERMS.

The appearance of small blue round cells and rhabdomyoblast-like cells on cervical cytology requires consideration of the possibility of small blue round cell tumors such as rhabdomyosarcoma. It is very difficult to make a diagnosis by solely relying on cell morphology, so the diagnosis can be made by incorporating the results of immunohistochemical testing of the cell blocks. Because the biological behavior of small, blue, round cell tumors often appears to be highly malignant, and their treatment and prognosis are different, accurate and differential diagnoses are very important [8]. The differential diagnoses by cytology included the following (Table 1): (1) Nonkeratinized squamous cell carcinoma (NSCC): Cells occur singly or in syncytial aggregates with poorly defined cell borders. Cells may be somewhat smaller than those of many HSIL, but display most of the features of HSIL. Nuclei demonstrate markedly irregular distribution of coarsely clumped chromatin with chromatin clearing. Nucleoli may be prominent. A tumor diathesis is often present. (2) Adenocarcinoma in situ of the cervix (AIS): Cells occur in sheets, clusters, pseudostratifi ed strips, and rosettes with nuclear crowding and overlap and loss of a well-defi ned honeycomb pattern. Single abnormal cells may be present but are uncommon. Cell clusters have a palisading nuclear arrangement with nuclei and cytoplasmic tags protruding from the periphery (“feathering”). Nuclei are enlarged, variably sized, and oval or elongated. Nuclear hyperchromasia with evenly dispersed, coarsely granular chromatin. Nucleoli are usually small or inconspicuous. Mitoses and apoptotic bodies are common. Background is typically clean (no tumor diathesis). (3) Adenocarcinoma of cervix: Cytologic criteria overlap those outlined for AIS, but may show additional features indicative of invasion. Abundant abnormal cells, typically with columnar configuration. Single cells, two-dimensional sheets or three-dimensional clusters, and syncytial aggregates. Enlarged, pleomorphic nuclei demonstrate irregular chromatin distribution, chromatin clearing, and nuclear membrane irregularities.The nucleoli are large, and cytoplasm is usually finely vacuolated. Necrotic tumor diathesis is common. (4) Small cell neuroendocrine carcinoma (SCNC): Small cell carcinoma is composed of relatively uniform small, cells with scant cyanophilic cytoplasm. The nuclei are angulated, hyperchromatic with granular or stippled chromatin and inconspicuous nucleoli. Background necrosis and mitotic figures are common. Currently, there is no consensus regarding the optimum management protocol for patients with cervical ERMS. Therefore, clinicians often follow the treatment guidelines for genitourinary primary ERMS. However, multimodal treatment appears to improve patient outcomes. This approach consists of a combination of surgical intervention, systemic chemotherapy, and/or radiotherapy for local control [7]. Since patients with ERMS are younger and often require fertility preservation, this is a major factor in treatment selection. When the lesion is localized to the cervix, the best treatment for preserving fertility is polypectomy and simple or radical trachelectomy combined with adjuvant chemotherapy [9, 10]. After resection, these patients should undergo adjuvant chemotherapy with vincristine, adriamycin D, and cyclophosphamide (VAC); these agents comprise the gold standard chemotherapeutic regimen [11, 12]. Our patient underwent adjuvant chemotherapy with vincristine, actinomycin-D and cyclophosphamide after receiving single-port laparoscopic-modified extensive total hysterectomy, bilateral salpingo-oophorectomy, pelvic lymph node dissection and abdominal aortic fat lymph node sampling. The patient’s computed tomography (CT) scan of the abdomen and pelvis after two cycles of chemotherapy did not show any evidence of disease.

**Table 1:** Differential diagnoses of ERMS

Tumor type	Age	Clinical symptoms	Cytologic characteristics	IHC (positive marker)
ERMS	children or adolescent	increased vaginal discharge or bleeding	Small blue cells, high nuclear to cytoplasmic ratio, and individual cells are somewhat tadpole-or ribbon-shaped. No nucleoli or nuclear division.	MyoD1 Myogenin Desmin
NSCC	adult female	contact bleeding or irregular vaginal bleeding	Cells may be somewhat smaller than those of many HSIL, but display most of the features of HSIL. Nuclei demonstrate markedly irregular distribution of coarsely clumped chromatin with chromatin clearing. A tumor diathesis is often present.	P63, P40, CK5/6
AIS	adult female	irregular vaginal bleeding or increased secretion	Cells occur in sheets, clusters, pseudostratifi ed strips, and rosettes with nuclear crowding and overlap and loss of a well-defi ned honeycomb pattern. Nuclei are enlarged, variably sized, and oval or elongated. Nuclear hyperchromasia with evenly dispersed, coarsely granular chromatin. Nucleoli are usually small or inconspicuous. Background is typically clean (no tumor diathesis).	CK8, CK18, P16
Adenocarcinoma of cervix	adult female	contact bleeding or irregular vaginal bleeding	Abundant abnormal cells, typically with columnar configuration. Single cells, two-dimensional sheets or three-dimensional clusters, and syncytial aggregates. Enlarged, pleomorphic nuclei demonstrate irregular chromatin distribution, chromatin clearing, and nuclear membrane irregularities. Macronucleoli. Cytoplasm is usually finely vacuolated. Necrotic tumor diathesis is common.	CK8, CK18, P16
SCNC	older female	vaginal bleeding	Small cell carcinoma is composed of relatively uniform small, cells with scant cyanophilic cytoplasm. The nuclei are angulated, hyperchromatic with granular or stippled chromatin and inconspicuous nucleoli. Background necrosis and mitotic figures are common.	CD56, CgA, Syn, NSE

Abbreviations: ERMS, embryonal rhabdomyosarcoma; NSCC, nonkeratinized squamous cell carcinoma; AIS, adenocarcinoma in situ; SCNC, small cell neuroendocrine carcinoma; IHC, immunohistochemistry; CK5/6, cytokeratin 5/6; CK8, cytokeratin 8; CK18, cytokeratin 18; CD10, common acute lymphoblastic leukemia antigen; ER, estrogen receptor; PR, progesterone receptor; CD56, neural cell adhesion molecule; CgA, chromogranin-A; Syn, Synaptophysin; NSE, Neurone-specific enolase

## Conclusion

Cervical smears are an important means of cervical cancer screening. In addition to screening for cancer, screening for other rare tumors is possible with such a modality. Although ERMS is uncommon in older adults, the disease must be considered in the differential diagnosis when there is vaginal bleeding or a protruding mass. In cervical cytology, the morphology overlaps with that of other tumors, so the differentiation of rhabdomyosarcoma from cervical cytological examination is very difficult. The pathologist should be familiar with these cytological features and characteristic findings from cell block examination and IHC, allowing early and accurate diagnosis of ERMS for timely treatment and avoidance of unnecessary surgical biopsy.

## Data Availability

All data generated or analyzed during this case are included within the article.

## Abbreviations

ERMS: Cervical embryonal rhabdomyosarcoma
IHC: Immunohistochemistry
NSCC: Nonkeratinized squamous cell carcinoma
HISL: High-grade squamous intraepithelial lesion
AIS: Adenocarcinoma in situ of the cervix
